# Neuroimaging-based prediction of mental traits: Road to utopia or Orwell?

**DOI:** 10.1371/journal.pbio.3000497

**Published:** 2019-11-14

**Authors:** Simon B. Eickhoff, Robert Langner

**Affiliations:** 1 Institute of Systems Neuroscience, Medical Faculty, Heinrich Heine University Düsseldorf, Düsseldorf, Germany; 2 Institute of Neuroscience and Medicine, Brain and Behaviour (INM-7), Research Centre Jülich, Jülich, Germany

## Abstract

Predicting individual mental traits and behavioral dispositions from brain imaging data through machine-learning approaches is becoming a rapidly evolving field in neuroscience. Beyond scientific and clinical applications, such approaches also hold the potential to gain substantial influence in fields such as human resource management, education, or criminal law. Although several challenges render real-life applications of such tools difficult, future conflicts of individual, economic, and public interests are preprogrammed, given the prospect of improved personalized predictions across many domains. In this Perspective paper, we thus argue for the need to engage in a discussion on the ethical, legal, and societal implications of the emergent possibilities for brain-based predictions and outline some of the aspects for this discourse.

## Abstract 2

Advances in machine learning on neuroimaging data have opened up the possibility of objectively predicting individual traits like intelligence, personality, or clinical risks from brain scans. This Perspective article discusses the methodological and ethical challenges arising from these advances.

Many potentially life-altering decisions that are made about a person by someone else involve judgments about “internal” characteristics like intelligence, trustworthiness, or other mental traits, that is, aspects that are not directly observable for the judge. This makes judgments difficult and error prone. For example, a company may want to hire a manager who is strongly determined and also very open to collaboration. Naturally, all applicants assert that they have these traits, so whom to select? As another example, a judge needs to decide whether counseling during incarceration has reduced aggressive tendencies to a level that does not pose a risk for others.

Traditionally, such questions have been tackled by extended interviews and looking at potential discrepancies between self-reported characteristics and previous behavior. This not only limits objectivity because of examiner effects but will also be biased by the degree the interviewees can “sell themselves” (i.e., their impression management skills), curtailing the validity of such assessments. Recent advances in the application of machine learning and artificial intelligence (AI) toward the predictive analysis of brain imaging data, however, may induce a disruptive change of this situation. Several studies now suggest that not only age or gender but also complex mental traits such as intelligence [1], attentional abilities [2], altruism [3], or personality factors [4] may be predicted in individuals from brain imaging data. Notwithstanding heterogeneity of technical implementations (see the aforementioned papers and [5,6]), the approach can be summarized as follows: Structural or functional (resting-state) neuroimaging data as well as the target trait measure are collected in large samples comprising several hundred participants. After preprocessing and a representation of individual neurobiology as parametric values, a machine-learning model is trained to find a mapping from the imaged brain features to the trait of interest. Generalization of the model is then assessed by predicting that trait in previously unseen people, either in an independent sample or through cross validation, and comparing the predicted with the (known) actual phenotype (Fig 1).

**Fig 1:**
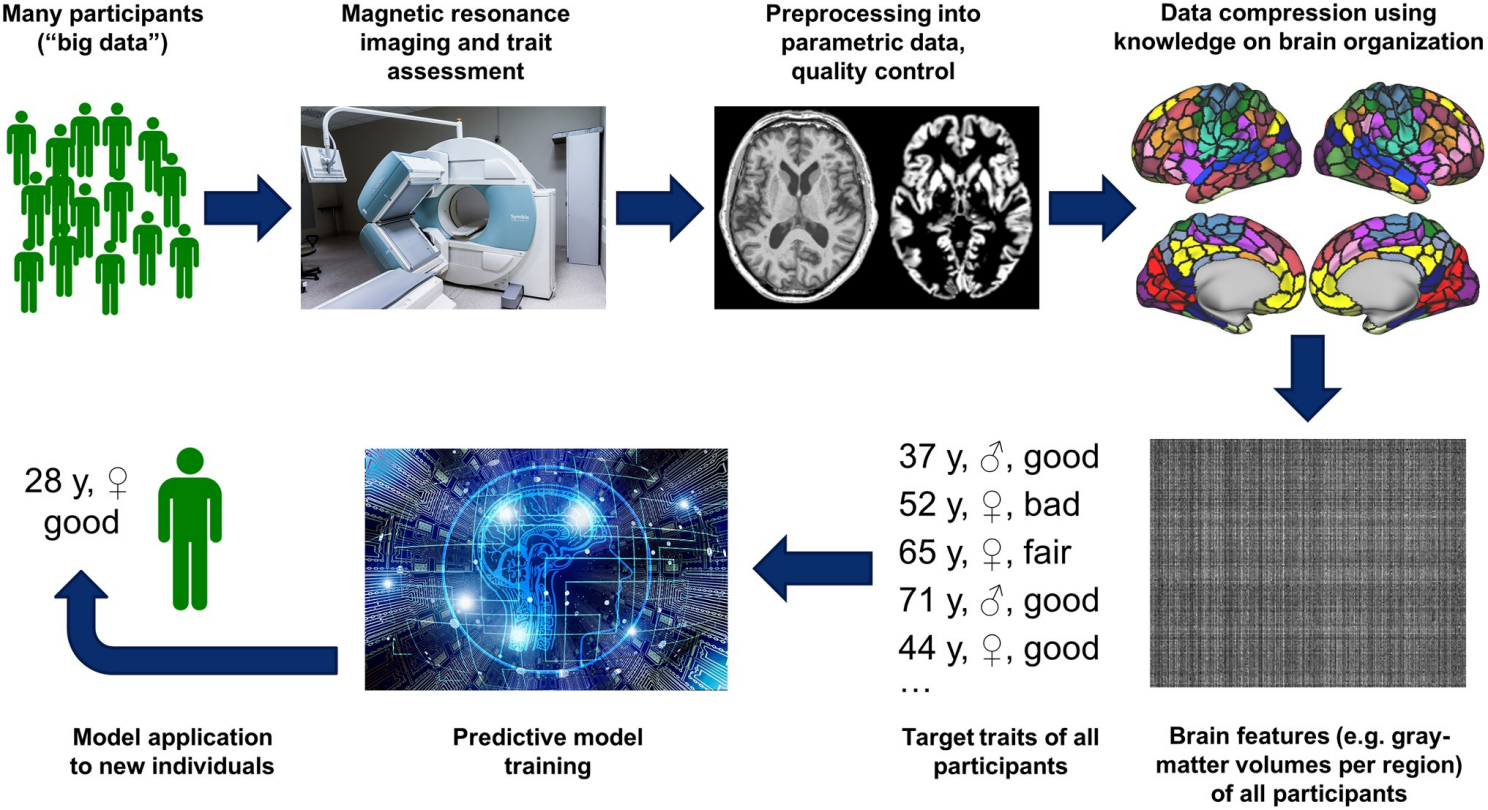
Schematic sketch of a pipeline for building brain-based prediction models for individual traits. To be read clockwise starting at the top left. *Parcellated brain hemispheres (top right panel) reproduced from* [7] *under a CC BY 4*.*0 license* (https://creativecommons.org/licenses/by/4.0/), *depicting results reported in* [8].

Have we as cognitive neuroscientists thus provided the basis for more objective and valid assessments of personal aptitudes, attitudes, and other mental characteristics, making everyone’s life better? If these methods are further developed and widely adopted, the entire society might benefit in many important aspects from improved evaluation and decision procedures that are devoid of implicit biases such as halo effects or other judgmental errors on the part of the observer. Besides being fairer and supporting equal opportunities for all, more valid assessments would engender more accurate matches between personal characteristics and contextual factors (e.g., specific therapies, job demands, or stressors), potentially enhancing health, life satisfaction, and productivity (compare with [9]). Or have we opened Pandora’s box and paved the way for an Orwellian future in which algorithms “know” our innermost features and dictate our potential choices in life? Ultimately, these questions can only be answered in the retrospective.

Although there has been increasing interest in the ethical and legal implications of neuroscientific progress since the early 2000s [10–15], neuroimaging-based prediction has only recently advanced to a degree that put it on the map for neuroethical discourse ([16]; see [17] for a review on using neuroimaging for violence prediction in legal contexts). At present, it seems appropriate to take a realistic look at the potential and limitations of these techniques and to identify issues for societal discussion.

First, a distinction must be made between scientific demonstrations of predictive power (e.g., a significant correlation between true and predicted traits in new participants) and algorithms that can be successfully used in real-life diagnostic or prognostic settings. To illustrate the point, a correlation of r = .70 would be considered a strong effect for group-level associations and, in fact, is probably the best that can currently be achieved for complex traits. However, it still explains only about half of the variance in the target variable. This exemplifies the frequently observed discrepancy between "statistically significant" and "practically relevant." It should be noted, though, that predictive models in neuroimaging are not only developed for personalized predictions, as focused on in this Perspective, but also with another goal in mind: to identify generalizable brain–behavior relationships. And for this purpose, finding substantial statistical associations like r = .70 would be considered highly relevant.

But how precise must an algorithm be to become relevant in applied settings entailing individual assessment? The answer obviously relates to the severity of the consequences of erroneous predictions. First of all, if a certain characteristic is rare, even a highly precise algorithm will produce many misclassifications and associated adverse consequences when used in large-scale evaluations (e.g., 90% accuracy will yield 100 errors in 1,000 cases screened). That said, would we accept 90% prediction accuracy in the context of a hiring decision? Most likely. But would it be acceptable for releasing an apparently rehabilitated child molester from detention? The majority answer might rather be “no” in this case.

In these scenarios, human evaluators also make mistakes and might barely fare better than (hypothetical) algorithms. Hence, do we impose higher demands for accuracy on AI? It seems so, but should this be the case? First of all, by using AI support, we aim to improve predictions and decision processes beyond the current human standards. Another part of the answer to this question, however, may be a lack of trust in AI because of its lack of discursive capacity: humans can present their thought processes and conclusions—even if partly confabulated post hoc because of the limits of introspection—which, in turn, allows others to integrate the decision with their own experience and knowledge and emulate and appraise the decision process. Algorithm-based predictions usually lack this (potentially spurious) explainability, which may constitute an obstacle to their broader societal acceptance. In addition, making life-impacting decisions might feel strange and discomforting or even illegitimate to many if it were solely driven by machine output, even if AI-based predictions were somewhat more accurate than human-made ones. How to weight human traceability and other “soft” features of the decision process vis-à-vis verifiably precise but unfathomable “black boxes” will most likely depend on the degree to which AI-supported algorithms reliably outperform human decision-makers.

Second, we need to acknowledge that the brain is not static and there is no one-way road from brain to mind (i.e., there is no unidirectional causal one-to-one mapping from brain activity to mental phenomena). Hence, we as human beings are not subject to a predefined fate coded in our neurobiology. This is particularly true when it comes to longer-term predictions, which may be of particular interest in many applications. Given the plasticity of the human brain, both the effects of agency (e.g., voluntary changes in lifestyle or approach) and outside influences may substantially impact the behavioral outcome of interest as well as the brain itself. For example, a job candidate may be predicted to be not well suited for a particular task but successfully works on herself to adapt to the challenges of the job, rendering the prediction invalid. Conversely, a criminal offender may have responded well to treatment and gets a very favorable prediction yet reverts to a problematic lifestyle after returning into previous social settings. How to accommodate such widening of the “predictive funnel” with time (i.e., the growing imprecision with increasing temporal distance to the predicted event) in neuroimaging-based predictions of behavior remains an open issue. This is also true for weather or traffic jam forecasts, to name just two examples of yet-unsolved prediction difficulties in complex dynamical systems in which the basic physical laws ruling the interactions of different factors are known—something that cannot be said of structure–function relations in the brain, let alone brain–behavior relationships. Noting that similar considerations hold for current expert assessments, we would argue that brain-based predictions should stimulate the respective discussions through quantitative estimates of predictive funnels.

Besides, the growing imprecision for temporally more distant events might be ameliorated by moving away from the binary nature of many prognoses (e.g., responsive versus not responsive to a given training, suitable versus not suitable for a particular job, or given versus not given to violence) toward time-sensitive continuous risk models as proposed by Matthew Baum [16]. This kind of probabilistic modeling has already been successfully adopted in other domains, such as forecasting rain and other weather conditions. Further, to accommodate the impact of contextual (nonbrain) factors like particular behaviors or social and environmental settings, pertinent data from smartphones and other wearable devices could provide complementary information to enrich and improve “neuroprediction” models.

Third, an oft-underestimated aspect in projections of future use is the discrepancy between technical and practical feasibility. The resources needed to assess hundreds or, more likely, thousands of people using neuroimaging are substantial, particularly when following these people longitudinally over months or years to observe a relevant (future) outcome. Furthermore, building practically relevant prediction models will likely require rather extensive imaging from each participant to achieve sufficient reliability despite the brain’s nonstationarity and potentially also multimodal data to cover various relevant aspects of neurobiology. For all this, highly cooperative participants are needed, also to achieve an appropriate level of data quality, as neuroimaging data are notoriously noisy and easily distorted or ruined by noncompliant behavior during scanning. Taken together, this is a huge challenge for developing as well as applying such models in real life, as the best model can only work if it gets all the input it requires. Last but not least, all these efforts will be futile if the quality of behavioral (psychometric) trait assessment is all but very high, as brain–behavior associations can never be closer—and thus, brain-based trait predictions never more precise—than is the level of reliability on either side ([18]; see also [19]). We need to keep in mind that traditional assessment procedures, although being the “gold standard” of trait measurement against which new prediction algorithms are evaluated, do not reveal the ground truth but come with their own shortcomings, as alluded to before.

Given these difficulties, how realistic are the promises and expectations outlined previously? In fact, the current picture is mixed: the initial prediction successes, which were too limited for real-life use, could not be markedly improved on by using larger samples ([20]; but see [21]). Also, even complex multivariate assessments like connectomic fingerprinting seem to be less individual and robust than expected [22]. This state of affairs likely results from a mixture of the aforementioned difficulties and other issues, some of which are beginning to be addressed, such as large-scale multimodal imaging and modeling. Furthermore, new markers of brain function and connectivity are likely to be identified, and prediction methods are going to be improved.

At any rate, as neuroimaging is rather costly, relative to other established or novel methods that may yield potentially predictive biomarkers (e.g., smartphones, ambulatory assessments, or electroencephalography), prediction based on neuroimaging data must be shown to clearly outperform competing approaches to justify its costs. From today’s perspective, given the remaining challenges, it seems unlikely that this kind of neuroprediction of mental traits will ever be universally applicable. A realistic expectation, though, might be its practical application in certain fields for specific questions, particularly when important issues are at stake for which other valid prognostic information is not available or otherwise obtainable.

The bottom line is that highly precise imaging-based prediction of mental traits in real-world scenarios requires substantial investments. Without these efforts, the ultimate potential of the outlined methods remains theoretical. It goes without saying that such challenges have far better chances to be met in settings with strong commercial or political interests of financially potent players. In such scenarios, however, conflicts of interest become an integral part of the process, and questions on permissibility arise. For instance, should an insurance company or a hospital group be allowed to train models on the data of their clients to predict future illness, even after obtaining individual consent to such data usage by their clients? Hardly anyone would disagree when the goal is to improve preventive care. But what if exactly the same data and results are used to cancel insurance coverage?

This illustrates an ethical issue previously discussed in regard to genetic data: the potential proliferation of inferential opportunities (compare with [23]). Data gained from conducting interviews, psychometric testing, or administering self-report inventories can mostly serve only the purpose it was collected for, whereas neuroimaging (like genetic) data, once collected, could be successfully submitted to a much broader number of predictive models, including those that were not yet thinkable when the data were acquired. Acknowledging the aforementioned aspects of plasticity, a brain scan obtained for an unrelated medical purpose could later be reused to assess, say, tendencies to violence and political extremism. Although this example is yet purely fictional, it still illustrates the potential uncontrollable misuse of brain imaging data. Considering how readily people are sharing genetic data with commercial companies, such a scenario could lead to a flourishing secondary market for predictive material. This obviously also applies to behavioral data, including verbal communication, obtained from mobile devices like smartphones and other wearables because of the broad scope of such data and the continuity of their collection, especially when combined with neuroimaging data as mentioned previously. Such considerations evidently lead to questions of data ownership, including the right to have data deleted, the limits of informed consent, as well as the weighting of personal and public interests. If and how neuroimaging data that could disclose personal information may be analyzed by current or future prediction algorithms is a question that only will grow in relevance when considering that through advanced data analysis, more and more types of data may yield predictive personal information in the future.

To conclude, it depends on us whether advances in the neuroimaging-based prediction of mental traits will move us closer to some form of utopia or drive us toward some Orwellian dystopia. Even if still a long, obstacle-strewn road ahead in any case, the core ethical and legal issues should be addressed now to avoid undesirable facts being established by individual stakeholders.
